# Erratum: The evolution of the brain, the human nature of cortical circuits, and intellectual creativity

**DOI:** 10.3389/fnana.2013.00010

**Published:** 2013-05-14

**Authors:** Javier DeFelipe

**Affiliations:** ^1^Instituto Cajal, Consejo Superior de Investigaciones CientíficasMadrid, Spain; ^2^Laboratorio Cajal de Circuitos Corticales, Centro de Tecnología Biomédica, Universidad Politécnica de MadridMadrid, Spain; ^3^Centro de Investigación Biomédica en Red sobre Enfermedades NeurodegenerativasMadrid, Spain

In the scale bar of **Figure 10**, there is an error. Instead of 10 cm it should be 20 mm. In addition, in the text and in the legend of **Figure 1**, there is a reference to a sculpture of Don Quixote present at the Museum of Modern Art in Mexico, a picture taken by the author of the present article. However, the author intended to say that this sculpture reminds him of a typical Don Quixote.

